# A Review of CNN Applications in Smart Agriculture Using Multimodal Data

**DOI:** 10.3390/s25020472

**Published:** 2025-01-15

**Authors:** Mohammad El Sakka, Mihai Ivanovici, Lotfi Chaari, Josiane Mothe

**Affiliations:** 1 Institut de Recherche en Informatique de Toulouse, IRIT UMR5505 CNRS, 31400 Toulouse, Francejosiane.mothe@irit.fr (J.M.); 2 Université de Toulouse, 31400 Toulouse, France; 3 Department of Electronics and Computers, Transilvania University of Brasov, 500036 Brasov, Romania; mihai.ivanovici@unitbv.ro; 4 Toulouse INP, 31000 Toulouse, France; 5 INSPE, Université de Toulouse, 31400 Toulouse, France

**Keywords:** convolutional neural network, smart agriculture, weed detection, crop disease detection, crop classification, yield prediction, water management, remote sensing, machine learning

## Abstract

This review explores the applications of Convolutional Neural Networks (CNNs) in smart agriculture, highlighting recent advancements across various applications including weed detection, disease detection, crop classification, water management, and yield prediction. Based on a comprehensive analysis of more than 115 recent studies, coupled with a bibliometric study of the broader literature, this paper contextualizes the use of CNNs within Agriculture 5.0, where technological integration optimizes agricultural efficiency. Key approaches analyzed involve image classification, image segmentation, regression, and object detection methods that use diverse data types ranging from RGB and multispectral images to radar and thermal data. By processing UAV and satellite data with CNNs, real-time and large-scale crop monitoring can be achieved, supporting advanced farm management. A comparative analysis shows how CNNs perform with respect to other techniques that involve traditional machine learning and recent deep learning models in image processing, particularly when applied to high-dimensional or temporal data. Future directions point toward integrating IoT and cloud platforms for real-time data processing and leveraging large language models for regulatory insights. Potential research advancements emphasize improving increased data accessibility and hybrid modeling to meet the agricultural demands of climate variability and food security, positioning CNNs as pivotal tools in sustainable agricultural practices. A related repository that contains the reviewed articles along with their publication links is made available.

## 1. Introduction

Smart agriculture involves the adoption of modern technologies and data-driven solutions to optimize resource usage and to enable real-time monitoring, leading to more sustainable and efficient agricultural practices. Referred to as Agriculture 5.0 (AG5.0), this approach leverages Artificial Intelligence (AI), the Internet of Things (IoT), and renewable energy sources to drive innovation in farming practices [1,2,3].

AG5.0 includes a wide range of applications that transform farming practices. One significant application is crop monitoring where AI-driven sensors enable the continuous observation of crop health and growth. Crop identification also helps in optimizing field management by distinguishing between crop types and assessing the composition of fields. Additionally, anomaly detection such as plant diseases and weeds becomes more efficient and precise than traditional solutions [1].

Traditionally, detecting anomalies in agriculture relied heavily on manual and visual inspection, where farmers assessed plant phenotypes—observable characteristics such as leaf color or blight spots—for signs of disease. This method, however, is time-consuming and labor-intensive, particularly as the global demand for agricultural products continues to rise with population growth. According to the Food and Agriculture Organization of the United Nations (FAO), “the global production of primary crop commodities reached 9.5 billion tonnes in 2021, increasing by 54 percent since 2000 and 2 percent since 2020” [4]. As agricultural demands grow, more efficient and scalable methods are needed to ensure the timely identification and management of crop anomalies. AI has become a critical tool in addressing these challenges, providing data-driven solutions that enable real-time decision-making and predictive capabilities based on plant phenotypes [5]. One of the most significant advances in this regard has been in the field of computer vision [6].

Computer vision enables computers to analyze and interpret visual data, transforming the way plant phenotypes are monitored and evaluated. Convolutional Neural Networks (CNNs) have become integral to computer vision, designed to detect and learn patterns from images [7]. Since the introduction of the groundbreaking CNN model AlexNet [8], which achieved an impressive 99% accuracy in the ImageNet classification challenge, CNNs have become essential tools for image analysis in many domains, including agriculture [9]. Recent developments have seen CNNs used as backbones for many generative AI models based on transformers [10]. In some cases, CNNs have even outperformed well-known transformers [11], which demonstrates that CNNs continue to evolve with new improvements and techniques, making them one of the most reliable computer vision algorithms for tasks such as image classification.

Similarly to other supervised machine learning models, CNNs undergo a training and testing process to learn and evaluate their performance. In the training phase, a CNN model processes a large amount of data through its layers using forward propagation, where it makes predictions [12]. These predictions are compared to true values and the model uses back propagation to adjust its parameters in order to improve its accuracy. After training, the model is evaluated on a separate testing dataset that it has not seen before. During this phase, the model’s ability to generalize to new data is tested, as an estimation to real-world performance [13]. Transfer learning can also significantly enhance this process, by allowing CNNs that have been pretrained on large and diverse datasets to leverage this knowledge to improve their performance on datasets specific to agriculture [14,15].

The input data play a crucial role in both training and evaluating models. The quality and diversity of data influences the model’s ability to learn and generalize in real-world scenarios with high accuracy [16]. Common types of image data include color images taken by cameras which consist of three channels (red, green, and blue, i.e., RGB) that show scenes the way the human eye would see it. However, images are not limited to the visible light spectrum. They can be captured in various other wavelengths, each providing unique information. For instance, infrared images are captured in the infrared part of the spectrum, radar images use radio waves, multispectral and hyperspectral images capture a wide spectrum of wavelengths across visible and invisible light, and other images can be heatmaps that visually represent data such as temperature or moisture levels. Images can be captured by different types of instruments, such as ground-based sensors embedded in soil or attached to plants to collect in situ data, handheld devices involving digital or multispectral cameras, aerial devices such as Unmanned Aerial Vehicles (UAVs) and satellites, or Unmanned Ground Vehicles (UGVs) [17,18,19,20]. A wide range of data types that are acquired differently exist, each offering a unique insight into various agricultural aspects. The data acquisition instruments also vary significantly, which could enhance the possibilities in agricultural monitoring and management. These advancements present notable research opportunities and have gained significant interest for the last few years. Although numerous reviews have addressed the use of AI in agriculture [21,22,23,24,25,26,27,28,29,30], there is still a lack of comprehensive discussion on data-related aspects and methodologies. This gap in the literature is significant, as effective AI-driven solutions are highly dependent on both data and methodology. To address this gap, we conduct a detailed analysis of recent articles published between 2018 and 2024.

More precisely, this review explores how CNNs are used to process various types of data in smart agriculture, while focusing on the specificity of each data type and their acquisition methods. Section 2 reviews relevant literature, including surveys and reviews on machine learning in agriculture. In Section 3, the methodology for gathering papers for this survey is described. In Section 4, a brief background on state-of-the-art models and evaluation metrics is provided. In Section 5, the papers are analyzed and categorized into five fields: weed detection, plant disease detection, crop classification, water management, and yield prediction. Section 6 offers a cross-application analysis and future perspective. Finally, a conclusion is made in Section 7 and an abbreviations table is provided. The reviewed articles can be found on a maintained github repository (https://github.com/MohammadElSakka/CNN_in_AGRI (accessed on 7 January 2025)).

## 2. Related Work

AI in smart agriculture is a multidisciplinary topic that is attracting growing interest from both researchers and engineers. Several recent reviews highlight the advancements and achievements where rapid progress in machine learning made it particularly influential in smart agriculture. For example, Liakos et al. [21] categorized machine learning methods by classifying them based on the issues they addressed. Their review analyzed over 40 studies that they categorized into four areas: crop management, livestock management, soil management, and water management. They concluded that methods such as clustering, decision trees (DTs), regression, neural networks, support vector machines (SVMs), and Bayesian models, are efficient in crop monitoring tasks such as yield prediction, disease detection, weed detection, crop quality, species recognition, tasks related to water, soil, and livestock management.

Kok et al. [22] reviewed the use of SVM in agriculture across the literature. They gathered 60 research articles that used SVM in addition to other machine learning and deep learning models, and then identified which model achieved the best performance. The studies they reviewed covered six key areas of agriculture: nutrient estimation, disease detection, crop classification, yield estimation, quality classification, and weed detection. Their findings indicated that SVM generally performed worse than Random Forest (RF) in certain areas and fell short compared to deep learning methods across all fields.

Kamilaris and Prenafeta-Boldú [23] analyzed 23 studies on how deep learning is used in farming and how methods are evaluated as well as how they compared to other approaches. They also conducted an experiment which goal is to detect missing vegetables in a sugar cane field. By using CNNs, they achieved 79.2% accuracy, which the authors considered low accuracy because of mislabeled images in their dataset.

Kamilaris and Prenafeta-Boldú [24] published a review on deep learning in agriculture, including insights on image preprocessing, image augmentation, and testing. A list of 14 image datasets that are publicly available was also provided.

While some reviews cover a global overview of AI uses in smart agriculture, others focused on more specific use cases. For instance, Liu and Wang [25] presented deep learning methods to detect diseases and pests in plants. Classification methods were detailed along with their advantages and drawbacks. Their findings indicate that some methods such as deep learning, especially CNNs, performed better than others, e.g., K-means, DT, SVM, and K-Nearest Neighbors (KNN). Another list of 14 image datasets of plant diseases and pests was also provided.

Saleem et al. [26] provided a list of studies that use CNNs in smart agriculture, in addition to visualization methods, e.g., segmentation maps, heatmaps, saliency maps, that are useful for analyzing decisions made by machine learning algorithms.

Kamarudin et al. [27] conducted a review on the use of deep learning in topics related to water stress such as evapotranspiration, water stress identification, soil moisture estimation, and soil water modeling. The review showed that deep learning models outperform traditional machine learning approaches in these applications. However, they also highlight that the application of deep learning in plant water stress assessment is still relatively new, and further research is needed to improve models.

Several other surveys focused on weed detection in crops using deep learning [28,29,30]. These surveys explored CNN model architectures and provided a list of publicly available datasets that are used in weed management.

## 3. Materials and Methods

**Figure 1:**
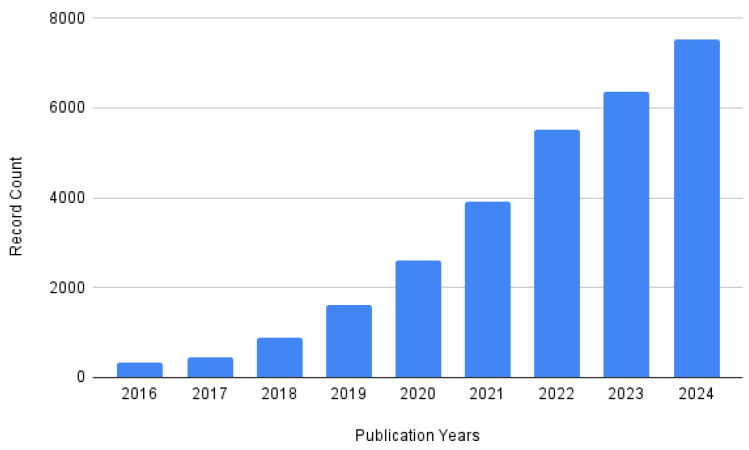
The evolution of the number of papers published every year on the Web of Science related to Convolutional Neural Networks, deep learning, or machine learning in agriculture.

**Figure 2:**
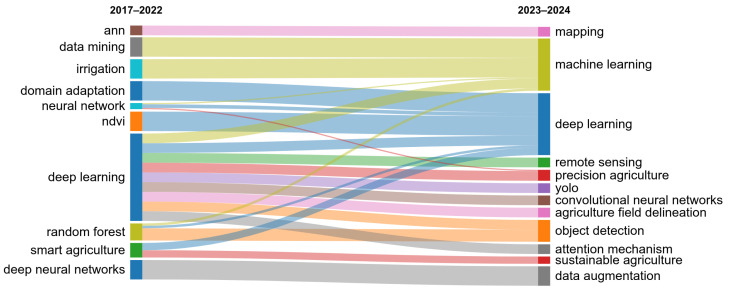
Authors’ keywords from all documents retrieved through the search query on the Web of Science, visualized using the thematic evolution function of the bibliometrix R package (version 4.3.0).

To conduct the literature review, keywords such as “Convolutional Neural Networks”, “Deep Learning”, and “Agriculture” were used to identify relevant papers published in conferences and journals. The analysis was conducted in two phases. First, an automated analysis was performed by querying the Web of Science database. Although this approach may introduce some bias, it provides a valuable overview of the field. A general query, “(convolutional neural network OR deep learning OR machine learning) AND agriculture”, resulted in 29,774 documents from which we kept 24,727 articles and 3396 proceeding papers. Then, by restricting the selection to English papers only, the results contained a total of 27,788 documents. Notably, the number of publications in this field began to increase significantly in 2018 (Figure 1). Papers on these topics were distributed across numerous venues. By focusing on venues that published at least five papers, we identified 733 papers on which we conducted an automatic bibliometric analysis. Figure 2 shows the authors’ keywords evolution. During the initial period between 2017 and 2022, non-deep learning methods were among the prominent methods but their prevalence has decreased since 2023. Instead, keywords such as “convolutional neural networks” and “yolo” became more prominent.

**Figure 3:**
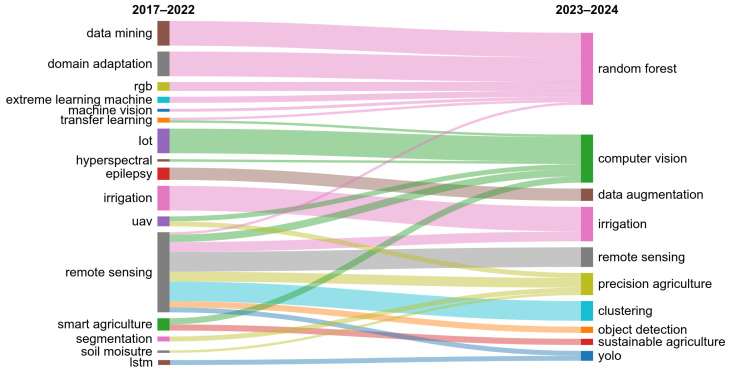
Authors’ keywords from all documents retrieved through the search query on the Web of Science, after excluding terms related to the search query, visualized using the thematic evolution function of the bibliometrix R package (version 4.3.0).

To gain deeper insights, we excluded terms that were related to the initial search query such as “convolutional neural networks”, “deep learning”, and “machine learning”. This revealed other notable trends, including the continued relevance of techniques such as RF, and the growing interest in sustainable and precision agriculture practices and modern topics related to irrigation (Figure 3).

In addition to the automatic analysis, we manually reviewed a subset of papers. The primary databases searched for this purpose included Google Scholar, the Web of Science, and IEEE Xplore. Articles were selected based on their relevance to the topic and the diversity of approaches they presented, ensuring a broad perspective on the application of CNNs in agriculture. The reviewed articles focused on one of five main applications in smart agriculture:

- **Weed detection**: Identifying and detecting weeds to enhance removal practices.
- **Disease detection**: Early identification of crop diseases through image analysis to minimize damage.
- **Crop classification**: Classifying different crop types for better field management.
- **Water management**: Monitoring water and moisture levels and optimizing irrigation practices.
- **Yield prediction**: Using visual and environmental data to predict crop yield more accurately.

Table 1 summarizes the number of papers that we reviewed for each field. The number of collected papers for each category varies depending on the variety of methods and techniques employed across the studies.

## 4. Background

CNNs have emerged as a powerful tool in image processing, achieving high performance across various fields from visual data. Since that progress, CNNs have transformed the way computers interpret visual information [12]. Originally developed for feature extraction from images for tasks such as image classification or segmentation, CNNs became widely used across domains requiring high accuracy in computer vision [31,32]. Their success can be attributed to their multilayer structure, where convolutional layers capture spatial features from images [12]. CNNs are typically evaluated using standard performance metrics, such as accuracy and precision, to quantify their effectiveness in tasks such as image classification [33]. Each of these metrics provides a different perspective on model performance. For instance, precision indicates how well the model correctly predicts positive predictions. Other metrics, such as Intersection over Union (IoU) are also often applied in image segmentation and object detection to evaluate the overlap between predicted and ground truth areas [34]. Key metrics that are often used in the studied literature for evaluating CNN models are presented in Table 2.

Table 3 presents an overview of widely adopted CNN architectures such as AlexNet [8] and ResNet [35]. Various state-of-the-art architectures have different designs suited to different tasks. For example, AlexNet was one of the earliest models to achieve high accuracy on image classification, while ResNet introduced a solution to the vanishing gradient problem in deep models. CNN architectures continue to evolve with enhancements, allowing them to have better feature extraction capabilities in order to become more efficient.

**Table 2:** Common performance metrics for machine learning models. This table provides an overview of standard metrics frequently used to evaluate model performances. Each metric includes its formula and a brief description.

Metric	Formula	Description
Accuracy [36,37,38]	$\mathrm{Accuracy}=\frac{Correct\;predictions}{All\;predictions}$	Ratio of correct predictions to total predictions.
Precision [39]	$\mathrm{Precision}=\frac{TP}{TP+FP}$	True positives over predicted positives; accuracy of positive predictions.
Recall [40]	$\mathrm{Recall}=\frac{TP}{TP+FN}$	True positives over actual positives; ability to identify relevant instances.
F1-Score [40,41]	$F1=2\cdot \frac{\mathrm{Precision}\cdot \mathrm{Recall}}{\mathrm{Precision}+\mathrm{Recall}}$	Harmonic mean of precision and recall; balances both metrics.
Mean Average Precision [42,43]	$\mathrm{mAP}=\frac{1}{N}\sum_{i=1}^{N}{\mathrm{AP}}_{i}$	Average of AP scores across classes.
Intersection over Union (Jaccard index) [44,45]	$\mathrm{IoU}=\frac{A\cap B}{A\cup B}$	Ratio of overlap area to union area; used in image segmentation and object detection.
Mean Intersection over Union [46]	$\mathrm{mIoU}=\frac{1}{N}\sum_{i=1}^{N}{\mathrm{IoU}}_{i}$	Average IoU across classes for multi-class evaluation.
Weighted Mean Intersection over Union [47,48]	$\mathrm{wIoU}=\frac{\sum_{i=1}^{N}w_{i}\cdot {\mathrm{IoU}}_{i}}{\sum_{i=1}^{N}w_{i}}$	mIoU with class weights $w_{i}$ to emphasize importance.
Processing Time [49]	T=timetakenforinference	Total time for model to process data and produce predictions.

**Table 3:** State-of-the-art CNN models. This table lists popular CNN models frequently used in the literature, along with links to their open-source implementations.

Model Name	Implementation URL (Accessed on 7 January 2025)
AlexNet [8,50,51,52],	https://github.com/amir-saniyan/AlexNet
CenterNet [53,54]	https://github.com/xingyizhou/CenterNet
DenseNet [55,56,57]	https://github.com/titu1994/DenseNet
Detectron2 [58,59]	https://github.com/facebookresearch/detectron2
EfficientDet [59,60,61]	https://github.com/rwightman/efficientdet-pytorch
EfficientNet [61,62]	https://github.com/lukemelas/EfficientNet-PyTorch
Faster-RCNN [36,39,63]	https://github.com/trzy/FasterRCNN
GoogLeNet [37,51,64]	https://github.com/conan7882/GoogLeNet-Inception
InceptionV3 [56,65,66]	https://www.kaggle.com/code/yasserh/inception-v3-implementation
Mask-RCNN [41,67,68]	https://github.com/matterport/Mask_RCNN
MobileNet [61,69]	https://github.com/cyrilminaeff/MobileNet
MobileNetV2 [56,61,70]	https://github.com/ShowLo/MobileNetV2
PSPNet [47,71]	https://github.com/Lextal/pspnet-pytorch
ResNet [35,56,72]	https://github.com/JayPatwardhan/ResNet-PyTorch
ResNeXt [56,73]	https://github.com/titu1994/Keras-ResNeXt
SEResNeXt [56,74]	huggingface.co/docs/timm/en/models/seresnext
SSD [75,76]	https://github.com/amdegroot/ssd.pytorch
SegNet [77,78,79]	https://github.com/vinceecws/SegNet_PyTorch
SqueezeNet [37,80]	https://github.com/cmasch/squeezenet
UNet [81,82,83]	https://github.com/zhixuhao/unet
VGG [51,84,85]	https://github.com/Lornatang/VGG-PyTorch
YOLO [86]	https://github.com/srebroa/awesome-yolo

## 5. Convolutional Neural Network Applications in Smart Agriculture

**Figure 4:**
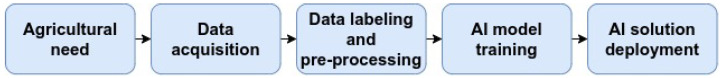
General workflow of smart agriculture. From identifying agricultural needs to deploying solutions, both data and models play a crucial role in developing effective solutions.

CNNs are designed for processing and analyzing visual data, especially images. Due to their efficiency in feature extraction from images, CNNs have been widely applied across various fields, consistently demonstrating high performances [31,87]. In the context of smart agriculture, CNNs have proven to be particularly valuable, aiding in decision-making processes by analyzing complex agricultural data. By being exposed to large datasets, CNNs are able to learn intricate patterns within images and generalize to new, unseen data, making them well-suited for a range of agricultural applications. Figure 4 illustrates the general pipeline used in smart agriculture, highlighting the various stages of the process. Depending on the specific agricultural needs, data is first acquired, preprocessed, and labeled. Then, an AI model is trained to address the agricultural problem. The ability of CNNs to efficiently analyze visual data has made them widely used in a range of applications in agriculture (Section 3). This section provides an analysis of these key applications.

### 5.1. Weed Detection

One of the most prominent applications is weed detection, where CNNs learn to distinguish weeds from crops. Weeds are undesirable plants in agriculture that compete with crops of interest for essential resources such as water, sunlight, or nutrients, and some types of weeds are toxic. They can also include remnants of previous year’s crops that re-emerge alongside current desired crops. If left unmanaged, they can significantly reduce crop yields and growth. Weed invasions are usually treated with herbicides, and very recently advanced techniques such as laser treatment [88]. Achieving precise herbicide control or laser treatment can involve computer vision techniques and more specifically CNNs for accurate weed detection. Weed detection is the identification and localization of weeds in agricultural fields, which can be achieved with CNNs in different approaches (Table 4).

**Image segmentation and object detection as main approaches.** A major challenge in weed detection is distinguishing weeds from crops, especially in complex environments where they may overlap or have similar visual features (e.g., color or spectral signatures). Two common machine learning approaches for addressing this challenge are image segmentation and object detection. In image segmentation, each pixel in the image is classified as weed or crop [47,48,78]. For example, Kamath et al. [47] showed that CNNs are efficient for weed segmentation in paddy crops, achieving a 90% weighted mean IoU with the use of the PSPNet architecture. Espejo-Garcia et al. [78] showed that using transfer learning to segment weeds from crops accelerated the training process and improved performance. Specifically, the authors trained a SegNet model on segmenting weeds from carrots and then applied the acquired knowledge to segment onions, and vice versa. Asad and Bais [48] achieved a frequency-weighted mean IoU of 98% in weed segmentation in canola fields using SegNet but noted difficulties in detecting weeds where they overlapped with crop leaves and confusion with plant stems because of high similarity between them.

**Table 4:** Summary of weed detection approaches in agriculture.

Category	Approaches	Purpose
Detection Techniques	Image segmentation (e.g., PSPNet, SegNet, UNet)	Classifies pixels as weeds or not (e.g., crops, background) [40,41,47,48,50,89,90,91,92]
	Object detection (e.g., YOLO, Faster-RCNN, Mask-RCNN)	Efficient at detecting and locating zones that contain weeds in an image [36,39,41,76,93,94,95]
Input Data Type	RGB	Most common data type in weed detection due to the different shapes of weeds that makes possible their identification [36,39,47,48,50,76,78,91,93,94,95,96,97,98,99,100,101]
	Multispectral	Improves weed detection performances [40,41,89,90,92,102]
	Vegetation indexes (e.g., NIR, NDVI)	Assists in distinguishing vegetation from non-vegetation [41,78,89,90,91,92]
Data Acquisition	UAV	Useful for aerial weed detection at different altitudes (1–65 m) [41,91,92,94,96,97,103]
	UGV	Efficient for close-range weed detection [40,50,89,98,99,101]
	Handheld devices (e.g., cameras, mobile phones)	Suitable for small scale weed detection [36,39,47,48,76,78,93,95,100]

On the other hand, object detection approaches aim to localize individual weeds or plants within a field using bounding boxes [36,59,76,93,96,104]. Zhang et al. [36] proposed a weed localization and identification method based on object detection, using Faster-RCNN. The authors integrated a Convolutional Block Attention Module that improves the efficiency of CNNs, achieving 99% accuracy at detecting and localizing several types of weeds and soybean seedlings. Chen et al. [76] used a local attention mechanism to effectively detect weeds in a sesame field outperforming other models, such as Fast-RCNN, SSD, YOLOv4, with a mean average precision of 96% and real-time detection speed. Jabir et al. [59] compared the performance of YOLO, Faster-RCNN, Detectron2, and EfficientDet at detecting weeds, and they found that YOLOv5 is a fast and accurate model that could be integrated to embedded systems for weed detection. To address the challenges of complex environments such as overlapping and small weeds, Wu et al. [96] improved the YOLOv4 model by modifying its backbone to include a hierarchical residual model, which improves small object detection. YOLO models are widely used in weed detection tasks due to their speed and efficiency in real-time detection, and the latest version, YOLOv11, further improves accuracy and processing speed [86,105,106,107]. Gao et al. [104] used both synthetic images and real images of weeds in sugar beet fields to train a CNN model that is capable of weed detection under complex situations, e.g., variation in plant appearances, illumination changes, foliage occlusions, and different growth stages. Generating synthetic images by cropping, zooming, flipping, and adjusting the brightness of real images allowed the model to better generalize on real situations.

**Multispectral data and vegetation indexes with RGB for enhanced weed detection.** While many studies used RGB images for weed detection or segmentation, others have explored a variety of data types, such as multispectral images or vegetation indexes [40,89,90,91,92,102]. Sahin et al. [89] compared different combinations of RGB, near infrared (NIR), and the Normalized Difference Vegetation Index (NDVI) [82] channels, as inputs to a UNet for weed segmentation. They concluded that green, NDVI, and NIR filtered with an edge-preserving Gaussian bilateral filter were the best input to their model. Moazzam et al. [90] showed that combining NIR to RGB images performs better than taking each one separately for weed segmentation.

**Data acquisition methods using UAVs, UGVs, and handheld cameras.** To acquire different types of data, sophisticated methods are employed, including UAVs [41,91,92,94,97,103], UGVs [39,50,98,99], and handheld cameras [95,100,101]. Ong et al. [97] compared the performance of a CNN with a classifier based on RF in weed detection using UAVs. To acquire data, the UAV was equipped with a camera of 20 megapixels resolution and a video resolution of 4K with 60 frames per second. The images were captured at two meters above ground level in JPEG format (RGB). The results showed that the CNN model outperformed the RF classifier by achieving an accuracy of 92% while being less sensitive to class imbalance in the dataset. Gallo et al. [94] demonstrated that it is possible to achieve an acceptable and realistic accuracy using high resolution RGB images captured from UAVs at 65 m above ground level. The employed YOLOv7 model outperformed other models, while also achieving real-time detection speed. Haq [103] compared several machine learning and statistical methods in weed detection using RGB UAV images taken at 4 m above ground level. They found that CNNs outperformed traditional methods like SVM, RF, DT, and AdaBoost, with an accuracy of 99%. Similarly, Osorio et al. [41] used a UAV equipped with a multispectral camera at 2 m above ground level. The images were captured in four spectral bands: green (500 nm), red (660 nm), red edge (745 nm), and NIR (790 nm). Osorio et al. [41] also added NDVI as a background estimator to help isolating vegetation from the background. Among the compared models, YOLOv3 and Mask-RCNN outperformed SVM, with F1-scores reaching 94%.

UGVs, or agricultural robots as referred to by some authors, were also used in weed detection [39,40,50,99]. Quan et al. [39] used a field robot to detect maize seedlings and weeds in a maize field under different weather conditions, e.g., sunny, rainy, cloudy. A camera mounted on the field robot captured RGB images of the field with different shooting angles, i.e., 0°, 30°, 75°. By using a Faster-RCNN model, the system achieved 97% precision in the detection of maize seedlings among weeds. Rasti et al. [99] captured field RGB images using a camera mounted on a UGV at 1 m above ground and found that integrating a scatter transform to a CNN could enhance model’s performances. Suh et al. [50] captured field images from a camera mounted at 1 m altitude on UGV with 0° for the angle of shooting. The study compared several CNNs with transfer learning on ImageNet and obtained the highest accuracy of 98% with an AlexNet-based model. Lottes et al. [40] used a field robot that captures images in RGB and NIR. The authors achieved more than 93% in F1-scores on weed segmentation using a CNN autoencoder with a spatio-temporal fusion module.

Cameras or sensors are not exclusively mounted on unmanned vehicles, but they can also be used as handheld devices. For example, many authors [93,95,100] used digital cameras to capture images in fields during different times of the day and under different weather conditions. Chen et al. [101] used mobile phones to build their image datasets for weed detection. Farooq et al. [102] used two multispectral cameras to detect several types of weeds. The first camera captures 16 bands between 460 and 630 nm, and the second captures four bands (green, red, red edge, NIR).

### 5.2. Disease Detection

CNNs play an important role in another agricultural application, which is plant disease detection. Plant disease detection is the process of detecting or identifying diseases in plants. Diseases impact crop health, leading to reduced yield and low quality crops. Left untreated, crop diseases may spread quickly in the fields, which directly affects food safety and agricultural products. By analyzing images of plants, CNNs can identify symptoms of diseases, which enables targeted and fast treatments as a part of crop management (Table 5).

**Table 5:** Summary of disease detection approaches in agriculture.

Category	Approaches	Purpose
Detection Techniques	Image classification (e.g., VGG, Inception, DenseNet, ResNet)	Efficient at identifying disease types in leaf images [51,56,85,108,109,110,111,112,113,114]
	Object detection (e.g., YOLO, CenterNet)	Used to detect areas in plants or fields that show disease symptoms [42,49,53]
	Image segmentation (e.g., Mask R-CNN)	Helpful at classifying diseased pixels in crops and leaves [68,115,116,117]
Input Data Type	RGB	Most commonly used for detecting visible symptoms [51,53,56,85,108,109,110,111,112,113,114,118,119]
	Multispectral images	Provides a high potential for early detection of diseases, without apparent symptoms [120,121,122]
Data Acquisition	UAV	Efficient for large-scale monitoring and real-time disease detection [42,49,53]
	Handheld devices	Used for close-range and on-ground images. Useful for quick data collection on fields and in controlled laboratory environment [51,56,85,108,109,110,111,112,113,114,118,119]

**Image classification for effective plant disease detection.** In the literature, plant disease detection is mainly solved using image classification, where models are trained to classify RGB images, into mainly two categories, e.g., healthy or unhealthy [51,56,85,108,109,110,111,112,113,114]. For instance, Thakur et al. [85] proposed a lightweight CNN architecture of 6 million parameters based on VGG and Inception that classifies plant diseases. Specifically, they employed their model on five datasets separately, with more than 100 crop diseases. The datasets consist of RGB images of several crop leaves that are either healthy or present multiple types of diseases. The model performed consistently well, reaching an accuracy of 99%. Kalbande and Patil [108] proposed a novel CNN model that uses several pooling techniques that include average pooling, max pooling, and global max pooling. Mixing pooling techniques aims to achieve a “smoothing to sharpening” approach in which average pooling and max pooling are applied to smoothen features extracted by convolutional layers, then global max pooling is applied to sharpen them. The method was applied to perform disease classification on images of diseased and healthy tomato leaves, reaching an accuracy of 95%. Panshul et al. [109] compared a CNN model to other machine learning and statistical algorithms, i.e., RF, SVM, Naive Bayes, Gradient Boosting, DT, KNN, and Multilayer Perceptron (MLP), in disease classification in potato plants. The models were trained and tested on potato leaf images. The CNN outperformed other methods by achieving 98% accuracy. Zhong et al. [110] proposed a light CNN model suitable for embedded systems. The CNN model uses Phish modules and light residual modules which improves feature extraction while reducing the size of the model. The proposed model outperformed other CNN models such as ResNet and VGG at tomato disease classification, achieving 99% accuracy while being lighter. Kaya and Gürsoy [111] proposed a novel deep learning method to identify plant diseases. Their approach involves applying image fusion between RGB images of plant leaves and versions of the same images with the background removed. After evaluating the method on 54,000 leaf images involving 38 classes, the model achieved 98% accuracy, outperforming state-of-the-art techniques. Furthermore, Ahad et al. [56] compared six state-of-the-art CNNs—DenseNet121, InceptionV3, MobileNetV2, ResNeXt101, ResNet152, and SEResNeXt101—in rice disease classification. Transfer learning from ImageNet could significantly improve classification accuracy, which rose up to 98% by SEResNeXt101. Similarly, Pajjuri et al. [51] compared AlexNet, GoogLeNet, VGG16, and ResNet50V2 in plant disease classification. VGG16 had the best performance, reaching an accuracy of 98%.

**As image segmentation or object detection.** While most studies approach plant disease detection as a classification problem, others consider it as an image segmentation problem [68,115,116,117]. For instance, Sharmila et al. [115] and Prashanth et al. [68] used a Mask-RCNN model to segment leaf images. The model successfully separated pixels that show symptoms of diseases from healthy leaves, reaching high performance levels. Shoaib et al. [83] used image segmentation as a preprocessing step before performing plant disease identification. The authors trained a UNet model to create a segmentation mask which effectively isolates leaves from the background. The isolated leaf images are then passed to an InceptionV1 CNN, which achieves 99% accuracy in determining whether the leaf is healthy or diseased. Kaur et al. [116] proposed a CNN model for tomato leaf disease segmentation. Their model successfully classified pixels showing plant diseases, e.g., early or late blight, achieving an accuracy of 98%. Similarly, Sharma and Sethi [117] used a CNN-based segmentation on wheat leaves to classify potential diseases.

Some authors also suggested object detection methods based on UAV imagery [42,49,53]. Liang et al. [53] proposed a CNN model based on the CenterNet architecture that detects diseases and insect pests in a forest. The method was applied on aerial images taken above a forest and it showed high accuracy and real-time speed, outperforming state-of-the-art methods. Wu et al. [49] used the YOLOv3 model with the complete IoU loss function which is optimized for object detection tasks. The authors used drone images of healthy and sick pine trees, and trained the model on detecting sick trees. The results showed an accuracy of 95%, with an average processing time of less than 0.5 s. Sangaiah et al. [42] employed a YOLO implementation in rice leaf disease detection from UAV images. The proposed model is lightweight and capable of being deployed on UAVs, while also having a high performance, reaching 86% mean average precision (mAP).

**Beyond RGB images.** RGB data is the most frequently used data type in plant disease detection across the literature due to the visual nature of symptoms. In addition to the visual aspect of plant disease symptoms, acquiring RGB images does not require a lot of material and is cheap. A significant portion of research thus focused on analyzing RGB leaf images. Nevertheless, recent studies are using non-visible imagery in plant disease detection, especially at an early stage, when symptoms are not visible yet [120,121,122]. Duan et al. [120] proposed a CNN model for early detection of blight using multispectral imaging. Blight in pepper leaves could be detected 36 h before visible symptoms start to appear. The CNN model achieved an accuracy of 91%, which demonstrates the feasibility of using multispectral imaging in early disease detection. De Silva and Brown [121] compared several deep learning techniques at detecting tomato diseases using multispectral images. Combining visible and NIR wavelengths achieved the highest accuracy, reaching 93%. It also showed that Vision Transformers (ViTs) outperformed CNNs, Hybrid ViTs, and Swin Transformers at this task. Reyes-Hung et al. [122] discussed the use of object detection methods based on YOLO to classify crop stress in multispectral images of potato crops. This study highlights the importance of using non-visible light, especially NIR and red edge, to detect plant diseases.

### 5.3. Crop Classification

Beyond identifying weeds and plant diseases, CNNs are also applied in classifying different crop types, which is essential for monitoring land use, crop rotations, and agricultural planning. Crop classification is defined as the identification and categorization of different crop types. Crop classification problems are solved using CNNs across the literature, using different approaches (Table 6).

**Table 6:** Summary of crop classification approaches in agriculture.

Category	Approaches	Purpose
Detection Techniques	Image classification (e.g., CNN-RNN-LSTM, CNN,)	Efficient at classifying images of leaves, plants, or fruits [123,124,125]
	Image segmentation (e.g., 1D-CNN, 3D-CNN, ViT, Recurrent CNN, HRNet)	High performances on classifying each pixel into the corresponding crop type [45,126,127,128]
Input Data Type	RGB images	Mostly used for leaf and plant classifications [52,124,125,129]
	Multispectral and hyperspectral images	Captures unique spectral signatures that are crop specific, assisting land cover crop classification [45,126,128,130,131]
	SAR data	Acquires detailed surface information. Useful to capture crop structure while being unaffected by weather conditions (e.g., clouds) [127,128,132]
Data Acquisition	Satellites (e.g., Sentinel-1, Sentinel-2, RADARSAT2)	Provides historical and periodical data for large-scale crop classification (e.g., land cover) [45,126,127,128,130,131,132,133,134]
	UAV	Captures high resolution aerial images that can be combined with satellite images to improve crop classification [52,128,129,131,134,135]
	Handheld devices	Close-range imaging for small scale classification [124,125]

**Classifying crop images.** Image classification approaches enable the classification of an entire image based on which crop it contains [123,124,125]. Gill et al. [123] proposed a CNN-RNN-LSTM model to classify field images of wheat. The proposed approach classified each image into a wheat variety class with an accuracy reaching 95%. Kaya et al. [124] compared the performances of CNNs using transfer learning and fine-tuning to classify leaf images of different crops. The results showed that transfer learning provides the best outcomes, reaching 99% classification accuracy. Lu et al. [125] proposed a six-layer CNN architecture to classify fruit images into nine classes. Their CNN model outperformed SVM, genetic algorithms, and feedforward neural networks, reaching 91% accuracy.

**Satellite-based remote sensing approaches.** Crop classification is not limited to classifying leaves or fruits but it can be employed at larger scales such as satellite images [45,126,127,130,132,133]. Yao et al. [45] used Sentinel-2 time series satellite images obtained on five dates to detect tea plantations. The authors proposed a combined model architecture between CNNs and Recurrent Neural Networks (RNNs) and compared its performance to methods such as SVM, Random Forest, CNN, and RNN. The RCNN model was trained on image segmentation by detecting pixels that show tea plantations. Their method outperformed various methods, by achieving an IoU of 79%. Rasheed and Mahmood [133] used Sentinel-2 time-series to identify rice crops among other classes (builtup, crops, rangeland, trees, and water), without the need of in situ data surveys. The NDVI was also adopted in addition to the multispectral inputs of Sentinel-2. A CNN approach was proposed and compared with various classical methods such as RF, SVM, classification and regression trees, Swin Transformer, HRNet, 2D-CNN, and Long Short-Term Memory (LSTM). Deep learning approaches outperformed traditional machine learning ones. Overall, the suggested approach achieved the highest performance, reaching 93% accuracy. Kou et al. [130] used Sentinel-2 multitemporal satellite images as input features and ground labels from a survey as output features for crop classification. Their proposed CNN outperformed RF, achieving high capabilities of generalizing over temporal data. Farmonov et al. [126] used hyperspectral images acquired using a spectrometer (DESIS) mounted on the International Space Station. While DESIS images had 235 spectral bands, ranging between 400 and 1000 nm, only 29 bands were selected based on their importance. The authors proposed a method based on wavelet transforms, spectral attention and CNNs to correctly classify pixels into several agricultural crops. Zhao et al. [132] used Sentinel-1 synthetic-aperture radar (SAR) time series for early crop classification. They used time series of VH+VV-polarized backscatter data as inputs to different models that they compared. The results showed that 1D-CNN outperformed RF, LSTM, and GRU-RNN, making it effective at classifying crops at an early stage using SAR satellite imagery.

**Fusion of Satellite and UAV Data.** Satellite images are often combined with UAV imagery for crop classification [128,131,134]. Yin et al. [134] proposed a ViT model based on 3D convolutional attention modules for crop classification tasks using multitemporal SAR data from UAV and satellites (RADARSAT2). The attention modules consisted of a polarization module and a temporal-spatial module to effectively learn features from temporal data and polarized data (SAR). The proposed model outperformed CNNs such as ResNet or 3DResNet, reaching 98% and 91% accuracy on UAVSAR and RADARSAT2, respectively. Li et al. [128] proposed a CNN model to segment crop parcels or objects in remote sensing time-series. The time-series consists of a combination of SAR data acquired by a UAVSAR and multispectral images by RapideEye. Labels were acquired by the United States Department of Agriculture that contains a wide range of data for agriculture. The authors showed that their method effectively outperformed other techniques.

**Applications of UAVs in crop classification.** UAV imagery is also used independently in many studies in crop classification [52,129,135]. Pandey and Jain [52] proposed an intelligent system based on CNNs and UAV imagery for crop identification and classification. An RGB camera was mounted on a UAV that flew at 100 m above ground to capture images. The authors compared the performance accuracy of the proposed CNN with other machine learning methods such as RF, SVM, and CNNs like AlexNet, VGG, and ResNet. Galodha et al. [135] used a UAV equipped with a terrestrial hyperspectral spectroradiometer to capture high resolution images of different crops. The authors compared several CNNs with a different number of layers and kernel sizes. CNNs with three or five layers achieved almost identical accuracy (87%) when 7 × 7 kernels were used. Kwak et al. [129] proposed a hybrid CNN-RF model for early crop mapping using limited input data. A CNN-RF model outperform CNNs and RFs because of its ability to leverage the advantages and strengths of both architectures for feature extraction and classification.

Crop classification has significantly benefited from remote sensing data, especially multispectral, hyperspectral and radar. The integration of advanced technology, such as sensors on UAVs and satellites, provides temporal data that is analyzed using CNNs in order to enhance crop monitoring and accurately identify crops.

### 5.4. Water Management

The next key application of CNNs in smart agriculture is the management of water resources. Water management focuses on optimizing water usage by monitoring irrigation, moisture levels, and potential droughts. By assessing the state of water resources, effective strategies can be developed in order to use water efficiently. Due to watering circumstances, general characteristics of plants vary significantly, e.g., the color, the shape, or the curvature. By exploiting these observable changes, several CNN approaches are used to address this topic, which support sustainable agricultural practices in the long-term (Table 7).

**Table 7:** Summary of water management approaches in agriculture.

Category	Approaches	Purpose
Detection Techniques	Image classification	High accuracy in detecting water stress, predicting droughts, and classifying different irrigation treatments [37,61,72,136,137,138,139,140,141]
	Regression	Accurately estimate soil moisture content, evapotranspiration, and groundwater content [142,143,144,145,146]
Input Data Type	RGB images	Effective for detecting visible changes (e.g., color, curvature) in plants under water stress [37,72,136,137,138]
	Multispectral and hyperspectral	Useful for early detection of water stress, even before visible symptoms [61,139,140,141]
	Vegetation indexes (e.g., NDVI, MSAVI)	Commonly used to assist in drought prediction and soil moisture estimation [140,143,147,148]
	Thermal	Helps in detecting water stress and estimating soil moisture [146,149]
	SAR	Beneficial in soil moisture estimation [143,147,150]
	Weather and in situ data	Effective in estimating evapotranspiration, groundwater, and soil moisture content [142,145,147,151]
Data Acquisition	Satellites (e.g., Sentinel-1, Sentinel-2, RADARSAT2)	Provides high spatio-temporal data, useful for soil moisture and irrigation mapping [143,144,145,147,150,152]
	UAV	Captures high resolution imagery, mostly used in water stress detection and soil moisture estimation [66,153]
	Handheld devices	Allows ground-level data acquisition for close-range water stress detection [37,137,138]
	Other sensors (e.g., tensiometers, thermometer)	Gathers data for better water management and ground truth labels [61,139,140,141,143,146,147,149,152]

**Color and spectral data for plant water stress detection.** Many studies use image classification techniques to differentiate between color images showing whether the plants suffer from water stress or not [37,72,136,137,138]. Kamarudin et al. [136] proposed a lightweight CNN based on an attention module for water stress detection. They considered images of plants that were subject to different water treatments, ranging from full irrigation to water deprivation. Their method outperformed state-of-the-art models, reaching 87% classification accuracy. Gupta et al. [72] and Azimi et al. [137] used images of chickpeas that went through three different watering treatments. Gupta et al. [72] found that ResNet-18 achieved 86% accuracy while Azimi et al. [137] achieved 98% accuracy using a hybrid CNN-LSTM model. Hendrawan et al. [37] compared four CNN models (SqueezeNet, GoogLeNet, ResNet50, AlexNet) at identifying water stress in moss cultures. They used RGB images of moss that received different water treatments, i.e., dry, semi-dry, wet, and soak. The authors found that the ResNet50 model achieved the best accuracy, reaching 87%. Zhuang et al. [138] used images of maize plants that were watered differently. The authors used a CNN feature extractor followed by an SVM classifier to classify images into categories ranging from drought stressed to well watered. Their method achieved a balanced performance between classification time and accuracy.

Color images are advantageous at detecting water stress in plants showing observable symptoms such as change in color or curvature. However, other authors used multispectral and hyperspectral images with the goal of identifying water stress earlier and with less observable symptoms [61,139,140,141]. Kuo et al. [139] used a hyperspectral spectrometer on tomato seedlings in order to detect early drought stress with the absence of visible changes. The authors proposed a 1D-CNN based on ResNet’s residual block and Grad-CAM that achieved 96% accuracy, outperforming other methods, while also minimizing computation and data collection costs. Spišić et al. [140] analyzed multispectral reads to detect water stress in maize canopies. SVM, 1D-CNN, and MLP achieved comparable performance, with trade-offs between performance and detection speed. Kamarudin and Ismail [61] compared several lightweight CNN models at drought stress identification in RGB and NIR plant images that went through different water treatments. Among MobileNet, MobileNetV2, NasNet mobile, and EfficientNet, EfficientNet achieved the best performance, reaching an accuracy of 88%. Zhang et al. [141] also compared different algorithms at detecting water stress in tomato plants using the visible and NIR spectrum and cloud computing. The MLP and one-vs.-rest classifier outperformed 1D-CNNs at processing 1D spectral data.

**Complementary data sources.** Other studies used thermal and weather data for water stress detection due to its correlation with factors like temperature, humidity, soil moisture, and evapotranspiration rates [142,146,149,151]. Li et al. [149] used thermal imagery acquired with a thermal camera along with RGB images of rice leaves that had different levels of water stress. The proposed method demonstrated that using CNNs to extract features from background temperature, along with plant thermal images, improved classification accuracy. According to the authors, this is due to the importance of air temperature, which directly relates to plant temperatures. Sobayo et al. [146] used thermal imagery to estimate soil moisture. They proposed a CNN-based regression model that generalized well over three farm areas, while outperforming traditional neural networks. Nagappan et al. [142] used weather data in order to estimate evapotranspiration in the aim of irrigation scheduling. The authors used data that includes wind speed, and max/min temperature as inputs, and evapotranspiration as labels. The authors demonstrated the effectiveness of a 1D-CNN to analyze 1D time series data. Afzaal et al. [151] compares several techniques for groundwater estimation using stream level, stream flow, precipitation, relative humidity, mean temperature, evapotranspiration, heat degree days, and dew point temperature. The study suggested that Artificial Neural Networks, MLP, LSTM, and CNNs were efficient at groundwater estimation, with MLP and CNN slightly outperforming other algorithms. Vegetation indexes were also used to assist in water stress identification [140,143,147,148]. For instance, Chaudhari et al. [148] showed that it is more common to have good results when using NDVI for drought prediction. Similarly, Spišić et al. [140] and Ge et al. [147] used NDVI in water deficit detection and soil moisture estimation, respectively.

**Satellite imagery for water stress and soil moisture monitoring.** While fixed optical cameras and weather variables are widely used in the literature, satellites showed to also be beneficial [143,144,145,147,150,152]. Liu et al. [143] used a combination of Sentinel-1 radar and Sentinel-2 optical satellite data in order to retrieve soil moisture in farmland areas. The authors used dual polarization radar (VH, VV), elevation and local incidence angle, polarization decomposition features (H,A,α) from Sentinel-1, and several vegetation indexes (NDVI, Modified Soil Adjusted Vegetation Index (MSAVI), Difference Vegetation Index (DVI) [82]) computed from Sentinel-2’s red and NIR bands, as inputs to different algorithms that were compared. The study showed that a regression CNN outperformed support vector regression (SVR) and generalized regression neural networks, and that MSAVI had the strongest correlation with soil moisture content, followed by NDVI, then DVI, due to the influence of MSAVI by both vegetation and soil. Bazzi et al. [150] used both Sentinel-1 and Sentinel-2 time series for tasks related to water management in agriculture. By using Sentinel-1 VV and VH polarization and red and NIR bands from Sentinel-2 and their derived NDVI, the authors compared different algorithms for mapping irrigated areas. They found that the CNN approach achieved 94% accuracy, outperforming RF. Ge et al. [147] compared several algorithms at estimating soil moisture from satellite observations. Their data included radar data from SMOS and ASCAT satellites, as well as NDVI retrieved from MODIS NDVI product MYD13C1. Here, CNNs can perform better than tradition neural networks in soil moisture retrieval from temporal satellite observations. Hu et al. [145] used microwave data from the Aqua satellite for soil moisture retrieval using regression. Again, a regression CNN performed better than SVR, while also being significantly faster.

**UAV approaches for soil moisture and water stress detection.** UAVs were used as well in different water management applications [66,153]. For instance, Wu et al. [153] proposed a method based on UAV remote sensing and deep learning for soil moisture estimation in drip-irrigated fields. Kumar et al. [66] employed a UAV-based technique using RGB images for water stress identification. The authors proposed a framework to identify different levels of water stress in a maize crop field. RGB images captured by a camera on a UAV were used to train different models. The proposed CNN outperformed models such as ResNet50, VGG19, and InceptionV3, achieving 93% accuracy. The authors used multimodal and multitemporal UAV imaging that captures RGB, multispectral, and thermal infrared wavelengths and showed that CNN-LSTM has a higher accuracy than CNN and LSTM models.

In conclusion, water management in agriculture encompasses a wide range of applications, especially, drought and water stress detection, irrigation mapping, and soil moisture estimation. Studies consistently showed that it is possible to efficiently accomplish effective water management using CNN methods with different data types acquired from a variety of sources at different temporal and spatial resolutions.

### 5.5. Yield Prediction

In addition to water management, accurate yield prediction is essential for effective agricultural planning. Yield prediction refers to the process of forecasting the quantity of crops that will be harvested from a field at the end of its growing season. It is an important aspect in agriculture that helps farmers in planning and making decisions regarding resources, supplies, and market strategies. With the emergence of AI and advanced technologies in computer vision, research studies focused on finding methods to correlate past yield values with image data (Table 8).

**Table 8:** Summary of yield prediction approaches in agriculture.

Category	Approaches	Purpose
Detection Techniques	Image classification	Used in identifying crop growth stages, which correlates with yield [38,154,155,156,157,158,159,160,161,162,163,164]
	Image segmentation (instance and semantic segmentation)	Used for crop segmentation and maturity classification, which helps in crop counting and yield estimation [43,165]
	Regression	Most commonly used technique for yield prediction [154,155,156,157,158,159,160,161,162,163,164,166,167,168]
	Object detection	Applied for detecting individual crop heads, fruits, or plants [165,169,170,171,172]
Input Data Type	RGB images	Effective for identifying crop growth stages based on different visible traits [43,157,165,169,170,171,172]
	Multispectral and hyperspectral images	Useful for detecting crop health and predicting yield [38,154,157,158,159,160,161,162,163,166,168]
	Vegetation indexes (e.g., NDVI, SAVI, EVI)	Helps in biomass estimation [157,160,162,164,166,167,168]
	Thermal	Improves yield prediction performances when combined with spectral data [158,160,162,163]
	Weather and in situ data	Provides more features to help in yield prediction [154,162,168]
Data Acquisition	Satellites (e.g., MODIS, Sentinel-1, Sentinel-2)	Used for large-scale yield prediction based on multitemporal and historical data [155,157,158,159,160,161,162,163,164,167,168,173,174,175]
	UAV	Provides high resolution imagery for yield estimation [38,57,79,154,166,170,171,176]
	Handheld devices	Offers localized data for specific crops with limited coverage but effective for small farms [43,165,169,171,172]
	surveys and land cover	Mostly used as labels for ground truth [38,154,155,156,157,158,159,160,161,163,166,167,168]
	Other sensors (e.g., in situ sensors)	Provides weather and soil related measurements, assisting yield prediction [38,155,156,162,167,168]

**Advances in crop yield prediction through multimodal data through regression.** Predicting or estimating crop yield is often a regression problem because of the scalar nature of the predicted values [154,155,156,166,167,173]. Mia et al. [154] compared different setups for yield prediction. The authors used CNN-based methods with UAV multispectral imagery and a combination of monthly, weekly, or no weather data. The best results were obtained with weekly weather data that included precipitation, global solar radiation, temperature, average relative humidity, average wind speed, and vapor pressure data. Tanabe et al. [166] used UAV multispectral imagery for winter wheat yield prediction and showed that CNN models outperform conventional regression algorithms such as linear regression. They also demonstrated that using multitemporal data of different growth stages may not improve the prediction accuracy if the CNN is effectively implemented. The heading stage of growth was sufficient for accurate predictions. Morales et al. [155] also used regression CNNs for winter wheat yield prediction. The authors used remote sensing data that included nitrogen rate, precipitation, slope, elevation, topographic position index, terrain aspect, and Sentinel-1 backscatter coefficients and showed that the proposed CNN method outperformed other techniques such as Bayesian multiple linear regression, standard multiple linear regression, RF, feedforward networks with AdaBoost, and a stacked autoencoder. Terliksiz and Altilar [173] used deep learning to extract features from MODIS multispectral data and land surface temperature time series. The proposed model concatenated features extracted by a CNN branch for multispectral and temperature data, and an LSTM branch for past yield data. This method is simple and efficient at crop yield prediction using multimodal and multitemporal data. Zhou et al. [167] used a CNN-LSTM model for rice yield prediction using time series that include MODIS remote sensing that and several vegetation indexes (Enhanced Vegetation Index (EVI), Soil Adjusted Vegetation Index (SAVI) [82,177]), Gross Primary Productivity, temperature data, spatial heterogeneity, and historical yield data. The proposed model consisted of a CNN block for spatial feature extraction followed by an LSTM block for temporal feature extraction. The proposed method outperformed other CNN and LSTM methods. In another hybrid approach, Saini et al. [156] proposed a CNN-LSTM method for yield prediction. The proposed approach includes a CNN block that extracts relevant spatial features, followed by a Bidirectional LSTM for phonological information. The proposed method outperformed other similar research studies.

**Object detection and image segmentation for image-based yield prediction.** While regression problems were suitable for crop yield prediction, some studies had different approaches. For instance, object detection methods were used to detect individual crops or fruits in RGB images. These approaches allow to estimate yields by counting fruits and identifying their maturity level [169,170]. CNN models were also trained on detecting crop heads and fruits, i.e., wheat head, cotton bolls, apples, and therefore estimating crop yield [165,171,172]. Maji et al. [43] proposed a combined approach of object detection and image segmentation in yield prediction. The proposed method predicts wheat yield by detecting wheat spikes using bounding boxes in the first place, then classifying wheat pixels in the second step. They reported a mAP of 97%, overcoming difficult conditions such as overlapping and background interference.

Using semantic segmentation, Ilyas and Kim [46] proposed a CNN architecture for strawberry yield prediction. Their method classified strawberry pixels into three maturity classes, enabling the yield prediction of each class. Their approach outperformed different DeepLab architectures, scoring a mean IoU of 80%. Yang et al. [38] showed that it is possible to estimate the yield of corn plants based on their growth stage. The authors suggested a CNN-based method that classifies hyperspectral images of corn plants in the field into five growth stage. Classification accuracy reached 75% when combining color and spectral information.

**Aerial crop yield prediction using UAVs.** To efficiently predict yield in crop fields, having a complete view of the field is beneficial in many studies and using UAVs is very common in this domain, as they provide an aerial view of plants [38,57,79,154,166,170,171,176]. Bhadra et al. [57] proposed an end-to-end 3D-CNN that uses multitemporal UAV-based color images for soybean yield prediction. They demonstrated that 3D DenseNet outperformed 3D VGG and 3D ResNet. Increasing the spatio-temporal resolution did not necessarily improve model performance; instead, it added more model complexity. Yu et al. [176] compared CNNs with several machine learning algorithms in maize biomass estimation using drone images. A UAV equipped with both a digital camera and a multispectral camera was used to acquire data from the field. CNNs outperformed traditional models and combining multispectral with RGB data gave the best results. Li et al. [79] used UAV imaging and CNNs to estimate cotton yield from a low altitude (5 m). The authors used image segmentation using a SegNet model to classify cotton boll pixels, then they applied linear regression on the segmentation result in order to obtain the yield. The proposed model outperformed SVM and RF.

**Large-scale crop yield prediction with satellite data.** Satellite imagery has also been used in crop yield prediction studies, offering a broad perspective of the fields over long periods [155,157,158,159,160,161,162,163,164,167,168,173,174,175]. For example, Fernandez-Beltran et al. [168] used monthly Sentinel-2 multispectral images with climate and soil data to estimate rice yields. The authors proposed a 3D-CNN that can extract temporal, spatial, and multispectral features from images. The proposed method proved to be effective at yield estimation, while outperforming state-of-the-art 2D and 3D-CNNs. Qiao et al. [160] also proposed a 3D-CNN for crop yield prediction using satellite time series. They used a multispectral dataset (MOD09A1) and a thermal dataset (MYD11A2) from MODIS satellite; the proposed method outperformed competitive methods such as LSTM, SVM, RF, DT, or 2D-CNNs. Additionally, 3D-CNNs are often used to process temporal images, especially in satellite remote sensing, where data is acquired periodically and historical records are available [159,175,178]. In different approaches, hybrid CNN models were used for yield prediction from satellite images. Hybrid models include a combination of CNN and LSTM modules, making them efficient at processing spatial and temporal data [161,163,174]. Other studies compared different algorithms and models at yield prediction using satellite data. For instance, Huber et al. [158] compared XGBoost, CNN, and CNN-LSTM in yield prediction. The study was conducted using time series from MODIS multispectral (MOD09A1) and thermal (MOD11A2) data, along with meteorogical variables (precipitation and vapor pressure). The results showed that XGBoost can be efficient at yield prediction, while outperforming state-of-the-art deep learning methods. Kang et al. [162] compared Lasso, SVR, RF, XGBoost, LSTM, and CNN at yield at maize yield prediction using remote sensing time series. XGBoost outperforms other algorithms, especially LSTM and CNN when datasets involve a small feature space.

## 6. Cross-Application Discussion

Following the detailed analysis of different CNN applications in smart agriculture, this section discusses the common factors across these applications, focusing on data acquisition methods, data types, and the role of CNNs in improving agricultural practices.

### 6.1. Data Acquisition

Data acquisition plays a critical role in the successful implementation of AI solutions across various agricultural applications. Different methods are employed to capture data depending on the specific task, whether it involves weed detection, disease detection, crop classification, water management, or yield prediction. Each method meets the needs of the specific application in which it was used. For instance, digital cameras are suitable for tasks requiring high resolution imagery at a close range for visual monitoring. They are mainly used in disease detection and weed management, where capturing clear high-resolution images allows the identification of specific symptoms of plant diseases or the presence of invasive weeds. Also, digital cameras are often the low-cost and easy to use and deploy, making them accessible for all farmers.

In contrast, UGVs are innovative but underused tools that can navigate fields with minimal human intervention. They can be equipped with different types of advanced sensors and cameras that allow them to monitor fields. They have been particularly useful at detecting weeds because of their ability to capture close-up images of crops and their surroundings, allowing for very precise crop monitoring. However, their use in the literature has been relatively limited compared to UAVs, likely due to their higher operational and maintenance costs, and the potential for causing damage to plants or soil.

UAVs have become highly effective for agricultural monitoring by offering a flexible way to collect aerial data over large areas. They can capture high resolution aerial images, while giving farmers access to real-time data. Like UGVs, UAVs can be equipped with various sensors and cameras (e.g., thermal, multispectral) to gather detailed insights particularly useful in many fields of agriculture, especially disease detection, weed management, crop classification, and yield prediction.

Acquiring weather variables and in situ measurements is essential for agricultural monitoring in farms and fields. By collecting data from weather stations, remote sensing and IoT devices, users can obtain real-time information about environmental conditions. This type of data can be used to assist decision-making in smart agriculture and enhance productivity.

Satellites provide a broad view of the landscape, making them essential for large-scale monitoring. Satellite data is mainly used for yield prediction, crop classification, and water management due to its wide coverage and ability to capture changes over time. Moreover, satellites provide historical and periodic data, making them valuable for long-term analysis. With advancements in space technology, satellite imagery is improving in resolution and benefiting from shorter revisit times, allowing more accurate and timely insights. As a result, the integration of satellite data with modern agriculture technology enhances decision-making and optimizes practices by leveraging past data to predict future outcomes and assess current conditions across large areas.

**Figure 5:**
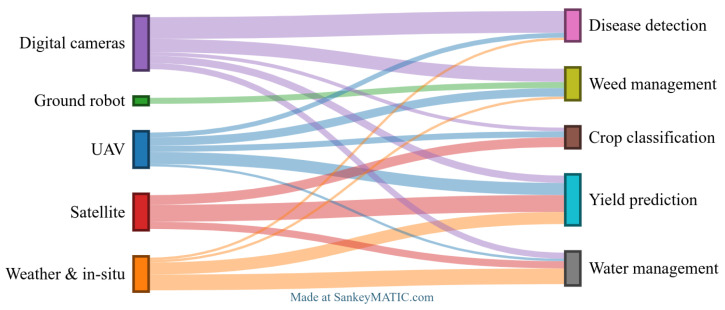
Data sources used in various fields in smart agriculture. This Sankey diagram illustrates the flow of data from various sources to different fields in agriculture. This diagram was created based on a review of the literature. Research papers were analyzed to identify data sources and the respective agricultural fields to which they were applied. Specifically, the terms on the left correspond to distinct data sources that were identified, while the terms on the right represent the different agricultural fields in which they were employed.

Figure 5 illustrates how each data acquisition method is applied based on the specific requirements of different agricultural fields. For instance, some fields like water management and yield prediction require complementary data sources (e.g., weather data and satellites), while others, such as disease detection, can typically be addressed using a single method of data acquisition (e.g., digital cameras).

### 6.2. Data Types

Different data acquisition technologies capture different data types that are suited to specific applications in agriculture.

RGB images capture standard color images, which are widely used for disease detection and weed management. Clear visual features provided in color images allow the detection of visible symptoms on crops, such as discoloration or unusual growth patterns, making them suitable for plant disease detection. Additionally, different shapes can be identified in color images, enabling to differentiate between plants and weeds, as well as classifying various types of crops. Moreover, the ability to detect objects in images helps in counting plants or fruits, thereby predicting crop yield.

In contrast, multispectral and hyperspectral images extend beyond the visible light spectrum. Their ability to capture multiple wavelengths in multispectral images or a continuous spectrum of hundreds of bands in hyperspectral images makes them valuable for applications where no visible features are present. This is particularly efficient in early plant disease detection or water stress. Healthier plants also lead to higher yields, so evaluating plant health also helps in predicting yield. Since different crops have distinct spectral signatures, multispectral and hyperspectral images are also effective for crop classification and weed detection. Additionally, they are used to compute vegetation indexes which provide more insights on crops.

Vegetation indexes are important information in agriculture that are calculated from multispectral imagery, mainly to assess plant health, biomass, chlorophyll content, or nutrient deficiencies. Indexes like NDVI and SAVI are essential for estimating the amount of vegetation in a given area, making them valuable for distinguishing vegetation from other objects. This ability provides additional knowledge that help in weed detection, disease detection, yield prediction, and crop classification. Moreover, indexes related to water, such as the Normalized Difference Water Index [179], are useful for managing water content and soil moisture in agriculture.

SAR images offer unique advantages for water management and yield prediction. SAR wavelengths can penetrate clouds and provide consistent data under any weather conditions, making them ideal for long-term and periodic monitoring, which is beneficial for predicting yields. Additionally, SAR is effective in measuring surface roughness and capturing information about soil moisture and plant structure, which also supports water related tasks and crop classification. This ability makes SAR a valuable data type, especially when combined with optical data.

Weather and in situ data-collection features used in agriculture provide information on environmental conditions that directly influence crops. These data are particularly important for assessing water stress and predicting crop yield, as they are both heavily affected by factors like temperature, precipitation, and humidity. Other important in situ variables that are measured in the field include pH, soil salinity, and soil moisture, all of which are significant in agriculture. By leveraging weather and in situ data, farmers can optimize their production by anticipating climate variability and natural disasters.

Thermal data also plays a crucial role in agriculture. In particular, surface temperature levels and variations over time provide valuable information to predict extreme conditions, which directly affect agricultural yields. Additionally, heatwaves can lead to droughts; therefore, monitoring thermal data can help in planning irrigation strategies.

**Figure 6:**
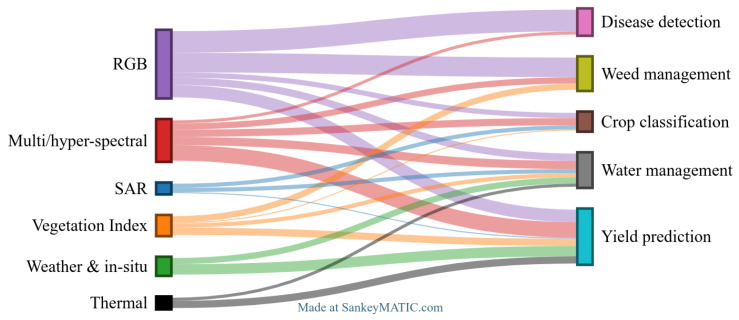
Data types used in various fields in smart agriculture. This Sankey diagram illustrates the various data types used in smart agriculture and their distribution across different agricultural fields. This diagram was created based on a review of the literature. Research papers were analyzed to identify data types and the respective agricultural fields to which they were applied. Specifically, the terms on the left correspond to distinct data types that were identified, while the terms on the right represent the different agricultural fields in which they were employed.

Figure 6 illustrates how each data type is used in different fields of agriculture. Some fields, such as disease detection and weed management, mainly do not require high-dimensional data and can be effectively addressed using simple RGB images. In contrast, other fields, such as water management, yield prediction, and crop classification, rely more on multimodal approaches to find solutions (e.g., hyperspectral and in situ or weather data).

### 6.3. CNN Relevance in Smart Agriculture

CNNs play an important role in smart agriculture by analyzing visual data that are found in various agricultural applications. These applications range from weed detection and disease detection to crop classification, water management, and yield prediction. CNNs solve agricultural challenges in different ways due to their capability of processing diverse data types collected from various instruments. The choice of CNN architecture and algorithm, however, is important to achieve high accuracy, real-time performance, and efficiency.

For tasks using RGB images, simpler CNN architectures are commonly used due to their ability to extract color-based visual features. More specifically, architectures such as AlexNet, as well as deeper models like VGG, or ResNet are efficient at feature extraction. This is especially true since versions of these models with pretrained weights on large datasets are available, specifically designed for three-channel inputs (RGB). For real-time object detection, e.g., on UAV systems, YOLO architectures are designed to be fast and accurate models, while Mask-RCNN and UNet are effective for pixel-level segmentation. Together, these approaches allow accurate and real-time agricultural monitoring using RGB images.

For more complex data such as multispectral and hyperspectral images, which consist of multiple wavelength bands, advanced architectures are more efficient to process both the spatial and spectral information simultaneously. In cases where time is an additional dimension, e.g., when collecting satellite data over growth season, spatio-temporal data processing becomes essential. While spatio-temporal data mainly comes from satellites, it can also come from UAVs or other sensors which offer temporal observations. This category of data also includes any data type, for instance, SAR or thermal. Three-dimensional CNN models are effective for handling both the spatial and spectral dimensions because they extend the traditional 2D CNN structure to process volumetric data. For example, 3D CNNs have been effective at processing aerial imagery, leveraging spatial, spectral, and temporal dimensions without relying on extensive feature engineering. This capability is advantageous in field-scale applications such as crop classification or yield prediction, reaching 98% accuracy in some cases [134]. In such cases, these architectures outperform simpler CNNs and traditional algorithms such as RFs and SVMs. Moreover, hybrid models such as CNN-LSTM or CNN-RNN are also well suited to extract temporal features from time series. These models combine CNNs for spatial feature extraction with LSTM or RNN layers to capture temporal dependencies, which improves predictions when the data involves changes over time. ViTs are another alternative that is particularly useful in tasks where long range dependencies in both space and time need to be captured. Unlike traditional CNNs, which rely on convolutions to extract local features, ViTs use a self-attention mechanism that allows them to determine the importance of each data feature relative to other features in that data [180]. When multimodal data combines images with tabular and scalar data, e.g., weather or in situ, additional approaches such as feature fusion are required.

Comparisons across the literature highlight the strengths and limitations of CNNs and other machine learning models. While CNNs consistently outperform traditional methods as SVMs and RFs in image-based tasks, models like XGBoost and RFs are often a better choice when structured tabular data is used. For example, in tasks using engineered features involving climatic variables, XGBoost have shown superior performance [158,162]. In contrast, CNNs and hybrid models such as CNN-LSTM, are more efficient in tasks requiring the processing of raw imagery and spatio-temporal data [66,156,167]. However, traditional methods remain more suitable when datasets are small or lack the complexity to justify the use of CNNs, as CNNs may overfit in such cases. Therefore, the optimal choice of model in smart agriculture depends directly on the nature and complexity of data, the specific task, and the available computational resources. Table 9 summarizes the strengths and limitations of different algorithms.

The feasibility of these models in real-world applications depends on factors such as speed, hardware requirements, and accuracy under practical conditions. Models designed for high speed and high accuracy such as Faster-RCNN and YOLO have shown a fast detection time on the order of milliseconds [53,76,94] on field images to few seconds on large satellite images [144]. Fast processing makes these models suitable for real-time detection in smart agriculture. Additionally, the lightweight nature of some models makes it possible to deploy them on embedded systems such as robots, field sensors or cameras, as they do occupy small amounts of memory to be deployed [42]. The high performance of CNNs reaching 99% accuracy under complex conditions, such as overlapping plants and varying weather conditions, further enhances their applicability in real-world scenarios [39,96].

### 6.4. Potential and Future Directions

Extensive research has been made on smart agriculture in the recent years and many studies have achieved high performances in various tasks. One of the future directions is to complete the gap between theoretical research and real-world applications, ensuring that smart solutions become available for everyday use in different agricultural settings.

Real-time performance in agriculture has been achieved in the literature. The future potential lies in enhancing the scalability of intelligent IoT systems in agriculture. In particular, further expanding the integration of IoT with cloud platforms for real-time data processing. Such a development is made possible by the improvements in sensor technology and GPU hardware.

Advancements in satellite technology offer shorter revisit periods and a higher spatial and spectral resolution. Improved spatio-temporal-spectral analysis opens up new opportunities for further advancement in remote sensing and smart agriculture. Additionally, the increasing accessibility of satellite data to a wider audience provides users (i.e., researchers, farmers, organizations) with valuable data for making informed decisions. As more users use this data, the potential for the application of satellite technology in smart agriculture continues to grow.

Large Language Models (LLMs) have also gained significant popularity due to their widespread use for text generation and their ability to process big amounts of data efficiently. Future research could focus on integrating text modalities to smart agriculture, enabling AI systems to deliver more comprehensive insight that would be context aware. For example, this integration would help users make informed decisions while taking into account different regulations and laws, especially with the recent advancements of LLMs in the legal domain [181] and the fusion of text with images [182].

AI in agriculture, like other fields, relies heavily on data, which raises privacy and security concerns. Technologies such as satellites and UAVs provide high-resolution images that can capture sensitive information about private properties and neighboring areas. Additionally, developing intelligent systems using such data could be exploited for competitive advantages, such as spying on strategies or gaining unauthorized information on crop yields or irrigation patterns. The use of IoT platforms and connected devices also adds another source of vulnerabilities that cybercriminals could exploit to access sensitive data or disrupt AI operations, leading to sabotage operations. Therefore, it is essential to direct research towards enhancing the security and privacy of AI-driven solutions in smart agriculture.

Furthermore, AI can assist in decision-making during the farming process, even at its earliest stages. Potential directions in smart agriculture could also focus on determining the probability and potentiality of agricultural lands before planting begins, leading to more strategic farming decisions.

Finally, Augmented Reality and Virtual Reality solutions could become critical tools in the future, especially with the ongoing progress in these fields. By providing visually enhanced data on farmlands, Augmented Reality can offer users a better understanding and management of their crops. Additionally, simulated environments, created through Virtual Reality can be crucial for experimenting various farming practices without consequences on real crops.

## 7. Conclusions

This review demonstrates the pivotal role of CNNs in smart agriculture. By analyzing a wide range of applications, namely weed detection, disease detection, crop classification, water management, and yield prediction, the study highlights the capability of CNNs to process multimodal data, such as color, multispectral, hyperspectral, and radar images. These advancements show how CNNs contribute to smart agriculture by revolutionizing farming practices.

When combined with advanced data acquisition techniques such as UAVs, satellites, or other IoT platforms, CNNs are effective for agricultural monitoring at different scales from close-range to field-scale. Also, by using 3D CNNs, handling more complex data that involves time series and volumetric data becomes possible. CNN capabilities are also brought to other techniques to form hybrid models such as CNN-LSTM or CNN-based ViTs that achieve better performance.

Significant progress in sensors, communications, and several other technologies, along with advancements in computer hardware, enhances the feasibility of integrating CNNs to develop more real-time, accurate, and real-world solutions.

Future research directions should focus on improving model scalability, making CNN models better integrated in large agricultural systems in real-world scenarios. Exploring how recent techniques, such as LLMs, can expand the capabilities of CNNs in smart agriculture is could result in new architectures that improve decision-making. Moreover, addressing challenges such as climate variability and data security is important to build trusted, sustainable, and safe prediction systems.

In conclusion, as agriculture moves toward Agriculture 5.0 and as AI continues to improve, CNNs play an essential role in offering smart farming practices to meet the demands of the future.

## Figures and Tables

**Table 1:** The number of selected papers for each field in smart agriculture. This number varies depending on the variety and diversity of methods employed across the studies.

Application	About	Number of Papers
Weed detection	Identifying unwanted plants within target crops	26
Disease detection	Diagnosing and assessing plant diseases to prevent their spread	23
Crop classification	Categorizing crop and plantation varieties	15
Water management	Managing water resources or detecting water scarcity	22
Yield prediction	Estimating future crop production levels	29

**Table 9:** Summary of different methods, their strengths, and limitations in the context of AI in smart agriculture.

Method	Strengths	Limitations
**CNNs (2D)**	Designed for image-based tasks, efficient at feature extraction for visual data, capture local spatial patterns, pretrained models are available	Risk of overfitting on small datasets, less suited for tabular or structured data
**3D CNNs**	Process spatial, spectral or temporal dimensions, effective for volumetric and hyperspectral imagery	High computational cost, more complex design
**CNN-RNN**	Combines spatial and temporal feature extraction, suitable for spatio-temporal data, e.g., CNN-LSTM	Increased complexity, high training time
**ViT**	Captures long-range dependencies and patterns, suitable for complex temporal or spatial feature extraction	Requires large datasets for training, computationally expensive
**Traditional machine learning**	Works well with small datasets or tabular data, fast training, easily interpretable results	Less effective for high-dimensional data and large datasets relative to other methods

## Abbreviations

The following abbreviations are used in this manuscript:

AG5.0	Agriculture 5.0
AI	Artificial Intelligence
CNN	Convolutional Neural Network
DT	Decision Tree
DVI	Difference Vegetation Index
EVI	Enhanced Vegetation Index
IoT	Internet of Things
IoU	Intersection over Union
KNN	K-Nearest Neighbors
LLM	Large Language Models
LSTM	Long Short-Term Memory
MLP	Multilayer Perceptron
MSAVI	Modified Soil Adjusted Vegetation Index
NDVI	Normalized Difference Vegetation Index
NIR	Near Infrared
RF	Random Forest
RGB	Red Green Blue
RNN	Recurrent Neural Networks
SAR	Synthetic-aperture radar
SVM	Support Vector Machine
SVR	Support Vector Regression
UAV	Unmanned Aerial vehicle
UGV	Unmanned Ground vehicle
ViT	Vision Transformers
